# Descriptive Genomic Analysis of Ampullary Carcinoma Utilizing the AACR Project GENIE Dataset

**DOI:** 10.3390/cimb47110932

**Published:** 2025-11-09

**Authors:** Samantha Martin, Blake Recupido, Elijah Torbenson, Beau Hsia, Marco Braaten, Abubakar Tauseef

**Affiliations:** School of Medicine, Creighton University, Omaha, NE 68124, USA; samanthamartin@creighton.edu (S.M.); blakerecupido@creighton.edu (B.R.); elijahtorbenson@creighton.edu (E.T.);

**Keywords:** ampullary carcinoma, genetic landscape, mutational characterization

## Abstract

**Background:** Ampullary cancer is a rare biliary tract cancer arising from one of the three epithelial tissues in the region. Leveraging a large patient-level genomic database, this study aims to identify, explore, and describe the genetic landscape of ampullary carcinoma and its implications. **Methods:** A retrospective analysis of ampullary cancer samples was conducted using the AACR Project GENIE database. Analysis of recurrent somatic mutations at large and between patient populations, and co-occurrence and mutual exclusivity of mutations was conducted, with a *p*-value < 0.05. **Results:** The most frequent mutations were identified as *TP53* (53.2%), *KRAS* (46.6%), and *SMAD4* (16.6%). Mutational differences were noted between sexes, White vs. Non-white groups, and histopathological subtypes. Significant mutual exclusivity was found between *KRAS* and *ERBB2.* Co-occurrence was observed in the *ARID1A* mutation with *KMT2D*, *ERBB2*, and *PIK3CA*; *CDKN2A* with the *SMAD4* and *KRAS* mutations; *TP53* mutation with the *CTNNB1* mutation; and *KRAS* co-occurred with an *APC* mutation. Reduced survival rates were seen in populations with the *TP53* or *KRAS* mutation. **Conclusions:** This study provides a detailed descriptive genomic landscape of ampullary carcinoma, highlighting frequent mutations between patient groups and the mutational burden of the DNA damage response pathway in ampullary cancer, laying important groundwork for the development of therapeutic targets and more individualized treatment regimens.

## 1. Introduction

The ampulla of Vater is composed of three histologically and physiologically distinct tissues: the common bile duct, the pancreatic duct, and a mucosal protuberance in the descending duodenum, and it is anatomically defined as the region where these three structures form a junction [1]. Ampullary cancer is a malignant neoplasm arising from the epithelium of any of these tissues. Most ampullary cancers are adenocarcinomas, with a small minority falling into the categories of papillary, adenosquamous, or mucinous carcinomas [2]. Further histopathological testing allows for categorization of the cancer to fall into one of two categories: pancreatobiliary, originating from the pancreatobiliary epithelium, or intestinal, originating from the intestinal epithelium [3]. The presentation of cancer most often includes jaundice, weight loss, and abdominal pain. Prognosis is highly dependent on the histopathological subtype, with the intestinal subtype having a 5-year survival rate of 61% and the pancreatobiliary subtype having a 5-year survival rate of 27.5% [4].

Ampullary cancer most commonly affects older adults, with an average age of diagnosis of 69 years [5]. It more commonly affects men compared to women (0.61 per 100,000 and 0.45 per 100,000, respectively) [6]. It has been noted in various studies that the incidence rate in both sexes has been increasing within the last 50 years [1,7]. The Caucasian population is the most frequently affected, but it has been shown that being Black has an independent association with worse survival rates [8]. Risk factors have been poorly investigated, but one study shows that a history of cholecystectomy or proton pump inhibitor use is associated with a higher risk of ampullary cancer [9].

The initial workup for suspected ampullary cancer requires an abdominal CT, an esophagogastroduodenoscopy with biopsy, and a colonoscopy, if not previously performed [10]. Once an ampullary carcinoma has been confirmed, imaging (CT and MRI scans) is pertinent for evaluating the extent of the tumor as well as assessing for any regional or distant metastases. Furthermore, histological subtyping and molecular testing of the biopsy are suggested [10]. If localized and resectable, a pancreatoduodenectomy (Whipple procedure) is performed, with postoperative adjuvant therapy. In patients with high-risk features (large primary tumor, regional lymph nodes, excessive weight loss, or extreme pain), neoadjuvant therapy is indicated [1]. There is little evidence suggesting a specific adjuvant, neoadjuvant, or even palliative treatment, leaving it up to the clinician. As it stands now, therapies are typically based on the historical subtype: intestinal is treated more similarly to colon cancer, and pancreatobiliary is treated more similarly to hepatocellular, biliary, and pancreatic cancer [4].

Ampullary carcinoma is characterized by a heterogeneous array of genetic alterations and pathways. The most frequently mutated genes are the tumor suppressor genes *TP53*, *SMAD4*, and *APC*, and the proto-oncogenes *KRAS* and *CTNNB1* [11,12]. The histopathologic subtypes carry differing frequencies of these mutations: The intestinal subtype carried more mutations in *APC*, *TP53*, and *KRAS*, similar to colorectal cancer, but the pancreatobiliary subtype showed a higher incidence of *KRAS*, *TP53*, and *SMAD4* mutations, similar to pancreatic cancer [13]. Furthermore, DNA mismatch repair deficiencies and microsatellite instabilities are more frequently found in the intestinal type [14,15]. However, the recent literature has shown that over one-third (40%) of diagnosed ampullary carcinomas are considered to be of the mixed subtype (showing characteristics of intestinal and pancreatobiliary) [16]. This warrants further studies on genetic markers in ampullary cancer at large, recognizing different markers in different population groups.

Despite the advances in the subcategorization of ampullary cancer, a descriptive genetic profile of the cancer remains incomplete. Identifying different genetic mutations, pathways, and drivers in ampullary cancer within different populations, sexes, and stages is essential for developing more robust and effective therapeutic interventions. Through leveraging publicly available data, this study aims to characterize the genetic profiles of ampullary cancer in different patient stratifications. Such characterization of the overall differences in the genetic landscape of ampullary cancer can be used to identify novel therapeutic targets, refine prognostic markers, and further develop therapeutic guidelines.

## 2. Materials and Methods

### 2.1. Genomic Database and Design

The AACR GENIE^®^ data-sharing project seeks to address precision cancer medicine using genomic data from 19 international cancer research centers. The approximately 229,000 sequenced samples in the registry were collected through heterogeneous platforms including targeted gene panels, whole-genome sequencing (WGS), and whole-exome sequencing (WES). Approximately 80% of the data was collected via targeted gene panels, 15% was collected via WGS, and 5% was collected via WES. Each sequencing platform achieved a different depth with targeted panels exceeding 500×, WES generating 150× coverage, and WGS fostering 30× coverage. Within the database, tumor-only sequencing contributed to 65% of the specimens while matched tumor-normal pairs contributed to the other 35%. Germline variant filtering was performed with the matched pairs.

For GENIE harmonization protocols to be followed, each participating institution utilizes its own version of the GATK (Genome Analysis Toolkit) and ANNOVAR software for variant detection and annotation, respectively, in accordance with the Genome NEXUS. Because multiple pipelines were in use during the consortium data submission period, software version numbers varied by institution; the submitted files were harmonized by the GENIE Coordinating Center as part of standard QA/QC procedures (see GENIE Data Guide v11.0-public). A certain subset of cancers within the database maintains available data on therapeutic responses and clinical outcomes. Treatment regimens, however, were not available for ampullary carcinoma. Additionally, because of the variability in collection from the participating institutions, it is possible that variations in bioinformatic pipelines may exist across the data. Genomic sequencing is performed with targeted panels recording up to 555 genes or whole-genome/exome sequencing.

This study was given exemption from institutional review board approval because of the use of the de-identified and publicly accessible American Association for Cancer Research (AACR) Project Genomics Evidence Neoplasia Information Exchange (GENIE)^®^ database by Creighton University (Omaha, NE, USA). The cBioPortal (v18.0-public) online software was used for data retrieval on 17 October 2025. This data included clinical and genomic information archived from 2017 through January 2025.

### 2.2. Data Collection and Processing

The cohort studied consisted of patients with a diagnosis of ampullary carcinoma obtained from a pathologic tumor process. Sample types were classified as primary (originating from the ampulla of Vater), metastatic and unknown in origin. Mutation differences between the primary and metastatic tumors were directly compared using a Chi-squared test.

Data obtained in this study included information on genomic differences (e.g., somatic mutations), clinical demographics (e.g., sex, race, and ethnicity), and histologic subtypes (e.g., not further classified, pancreatobiliary, intestinal, or mixed). Additionally, certain key genes were found to have a strong association with ampullary carcinoma (e.g., *TP53*, *KRAS*, *SMAD4*, *APC*). The targeted panels did not contain non-actionable genes, and the analysis does not include structural variants. Gene frequencies were calculated using the total number of patients in the cohort as the denominator, rather than the number of samples tested for each individual gene. This approach was used to avoid overestimating the frequency of genes that were only assayed in a subset of samples. Copy number alteration frequencies were calculated using the total number of samples profiled for that alteration as the denominator.

Further, the presence of copy number alterations (CNAs) was reviewed, exploring loss of heterozygosity (LOH) events in which homozygous deletions or amplifications occurred. The frequency of recurrent events was then additionally calculated. The AACR GENIE database then calculates the tumor mutational burden (TMB) as somatic mutations per megabase sequence normalized by panel size (e.g., somatic mutation total/2 for a 2 Mb panel). Linear regression models were used to adjust the data and estimate whole-exome sequencing (WES)-equivalent TMB. This information is available with request to GENIE. To correct for heterogeneity in the panel size and increase comparability, these models incorporate various factors, including panel size.

### 2.3. Statistical Analysis

Statistical analysis was performed using R/R Studio version 4.2 (R Foundation for Statistical Computing, Boston, MA, USA), with statistical significance defined as *p* < 0.05. Continuous variables are identified as means ± standard deviations (SD), and categorical variables are presented as frequencies and percentages. Chi-squared test was used for comparison amongst categorical groups, while continuous variables used normality and a two-sided Student’s *t*-test or a Mann–Whitney U test for direct comparisons. The Benjamini–Hochberg false discovery rate (FDR) correction was used for adjusting multiple comparisons. Exclusion criteria for analysis included samples with missing data.

Filtering criteria were used to select nonsynonymous variants (frameshift, missense, nonsense, and splice-site mutations) amongst the somatic mutations. The mutations must present with a variant allele frequency (VAF) of ≥5% and a sequencing coverage of ≥100×. Exclusion from analysis of synonymous mutations and variants of unknown significance was also performed. The GENIE harmonized mutation annotation format files additionally provided mutation calls with standardized annotations of variance amongst the 19 participating cancer centers.

To perform survival analysis, Kaplan–Meier curves were generated for each of the 15 most frequently mutated genes in the cohort. Each gene had two curves computed: one representing patients with a mutation in the gene, and the other representing patients with the wild type. Log-rank tests were calculated to test statistical significance (from the survival package, version 3.7.0). The GENIE database does not include a time of diagnosis variable, so the patients’ age at the time of sample sequencing was used as a substitute for time of diagnosis. The interval between the time of sample sequencing and the time of death (or last follow-up) was used to calculate survival time, retaining only patients who had a greater than zero survival time. Because some individuals have multiple samples, mutation counts for each gene were combined across all samples in each patient. Overall gene mutation frequency was determined on a per-patient basis by classifying a gene as mutated if the total count of alterations across all samples for a given patient was greater than one.

## 3. Results

### 3.1. Patient Demographics of Ampullary Carcinoma

Due to the limited sample size of ampullary carcinoma within sequenced cohorts, the initial demographic analysis combined primary, metastatic, and unknown tumor samples. Table 1 shows detailed demographic data. This study consisted of 478 samples from 466 patients. Of these patients, 260 (53.4%) were male, 217 (44.6%) were female, and 10 (2.1%) were unknown. In terms of race, the cohort comprised 40 (8.2%) Asian, 319 (65.5%) White, 32 (6.6%) Black, and 61 (12.5%) Other Identifying patients. Regarding ethnicity, 320 (65.7%) were non-Hispanic, 61 (12.5%) were Hispanic, and 106 (21.8%) were unknown/not collected. The race of 27 (5.5%) patients was unknown. All patients were 18 years of age or older. Of the samples, 303 (60.6%) were from primary tumors, 147 (29.1%) were from metastatic tumors, and 50 (10.0%) were unspecified.

### 3.2. Most Frequent Somatic Mutations and Copy Number Alterations of Ampullary Cancer

Table 2 summarizes the specific somatic mutations that most frequently occurred in this cohort. *TP53* mutations were the most prevalent, with notable frequencies in *KRAS*, *SMAD4*, and *APC*. Additionally, we identified recurrent copy number alterations (CNAs) in 342 samples. Loss of heterozygosity (LOH) events were prevalent, particularly affecting the tumor suppressor genes *CDKN2A* (*n* = 41; 12.4%), *CDKN2B* (*n* = 33, 10.3%), and the oncogene *MDM2* (*n* = 26, 7.9%).

### 3.3. Mutational Differences by Sex (777 Genes Tested in Total)

Stratification by sex revealed that some mutations occurred at higher frequencies either females or males. The mutations that occurred at a higher frequency in females vs. males were *FANCA* (*n* = 10 (5.35%) vs. *n* = 1 (0.43%), *p* = 3.86 × 10^−4^), *SF3B1* (*n* = 10 (5.13%) vs. *n* = 3 (1.25%), *p* = 2.29 × 10^−2^), and *ATRX* (*n* = 10 (5.35%) vs. *n* = 3 (1.29%), *p* = 2.21 *×* 10^−2^). The mutations that occurred at a higher frequency in males vs. females were *PREX2* (*n* = 2 (1.82%) vs. *n* = 11 (7.69%), *p* = 4.41 × 10^−2^), *CTNNB1* (*n* = 12 (5.41%) vs. *n* = 31 (11.61%), *p* = 1.64 × 10^−2^), and *RARA*, which exclusively occurred in males (*n* = 5 (2.18%), *p* = 6.92 *×* 10^−2^). Differences in mutations by sex are shown in Table 3. To assess comparability, weighted shared-test fraction (WSF) was calculated. For sex-based differences, all 6 of the tested genes showed strong overlap (WSF 0.93–0.96).

### 3.4. Mutation Differences by Race (765 Genes Tested in Total)

When stratified by race (White vs. Non-White, excluding not collected and unknown), The mutation differences found between the Non-White and White groups are shown in Table 3. To assess comparability, WSF was calculated. For race-based differences, *KRAS/TP53/SMAD*4 had acceptable overlap (WSF ~0.66). *IRS2/SOX9* were moderate (WSF 0.60–0.63); *LRP1B* was weak (WSF 0.24).

### 3.5. Co-Occurrence and Mutual Exclusivity Mutations (78 Gene Pairs Tested in Total)

Co-occurrence patterns were significant among genes that were frequently mutated. With statistical significance, *ARID1A* mutations co-occurred with the mutations *KMT2D* (*n* = 14, *p* < 0.001, *q* < 0.001), *ERBB2* (*n* = 10, *p* = 0.014, *q* = 0.118), and *PIK3CA* (*n* = 10, *p* = 0.014, *q* = 0.118). *RNF43* mutations co-occurred with mutations in *ARID1A* (*n* = 12, *p* < 0.001, *q* < 0.001) and *KMT2D* (*n* = 11, *p* < 0.001, *q* < 0.001). *CDKN2A* mutations co-occurred with the mutations *SMAD3* (*n* = 24, *p* = 0.016, *q* = 0.121) and *KRAS* (*n* = 50, *p* = 0.017, *q* = 0.121). The *TP53* mutation co-occurred significantly with *CTNNB1* (*n* = 26, *p* = 0.005, *q* = 0.100) and *KRAS* significantly co-occurred with *APC* (*n* = 45, *p* = 0.008, *q* = 0.106). The only mutually exclusive mutations found were *KRAS* and *ERBB2* (*p* = 0.022, *q* = 0.140), and *KRAS* and *ATM* (*p* = 0.041, *q* = 0.244).

### 3.6. Primary vs. Metastatic Mutation Differences (776 Genes Tested in Total)

This study comprised 301 primary tumor samples and 147 metastatic ampullary carcinoma cases. For genomic analysis of comparison, the samples from primary and metastatic tumors were included. For direct comparison of the mutations of primary vs. metastatic samples, the “unknown/NA” group was excluded. The *MDM2* mutation occurred more frequently in the primary tumors vs. metastatic tumors (*n* = 27 (10.76%) vs. 2 (1.54%), *p* = 8.052 × 10^−4^, *q* = 0.106) The mutations that were enriched in the metastatic samples were *RET* (*n* = 9 (6.16%) vs. 5 (1.65%), *p* = 0.0170, *q* = 0.472) and *TP53* (*n* = 91 (61.90%) vs. 156 (51.66%), *p* = 0.0420, *q* = 1).

### 3.7. Mutations Differences by Histological Sub-Category

We stratified the tumors by histologic subtype using the given database categories of not further classified (*n* = 288, 57.6%), pancreatobiliary (*n* = 159, 31.8%), intestinal (*n* = 38, 7.6%), or mixed (*n* = 15, 5.0%). The intestinal subtype had higher frequencies in the mutations *APC* (*n* = 20 (55.56%), *p* < 10 *×* 10^−10^, *q* = 7.84 *×* 10^−10^), *CTNNB1* (*n* = 9 (23.68%), *p* = 2.379 *×* 10^−3^, *q* = 0.0186), and *SOX9* (*n* = 10 (33.33%), *p* = 5.329 × 10^−9^, *q* = 8.16 × 10^−8^). The mixed subtype had notable frequencies compared to the others in *FAT1* (*n* = 4 (36.36%), *p* = 6.47 × 10^−4^, *q* = 5.823 × 10^−3^), *RUNX1* (*n* = 2 (16.67%), *p* = 3.208 × 10^−5^, *q* = 3.441 × 10^−4^), and *ERG* (*n* = 2 (18.18%), *p* = 1.213 × 10^−4^, *q* = 1.266 × 10^−3^). The pancreatobiliary subtype had notable frequencies in the *BAP1* gene (*n* = 7 (4.86%), *p* = 0.0757, *q* = 0.286). The mutation differences in the histological subgroups are summarized in the Supplementary Table S1. In total, 1487 genes were tested.

### 3.8. Mutations Associated with Reduced Survival

**Figure 1:**
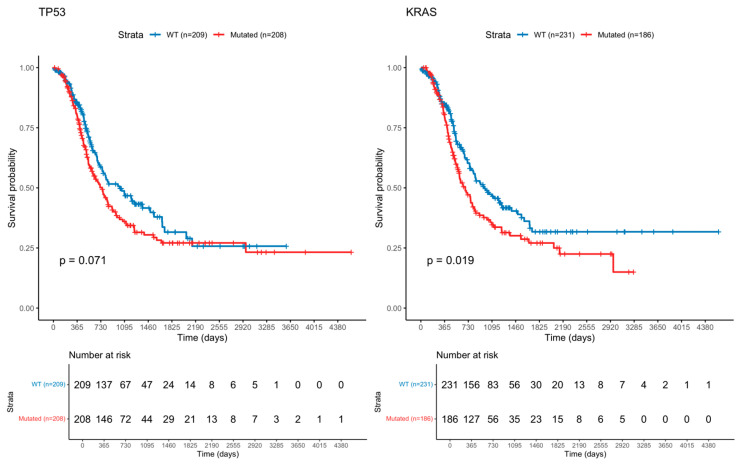
Kaplan–Meier Curve of Ampullary Cancer with *TP53* and *KRAS* mutation.

Survival analyses were performed for the 15 most frequently mutated genes, comparing patients with mutations to those with wild-type tumors. Genes showing significant differences with log-rank test are summarized in Table 4, and the corresponding Kaplan–Meier survival curves are presented in Figure 1.

### 3.9. Survival Analysis with Mutually Exlcusive and Co-Occurring Genes

Survival analysis was performed on several genes that were either mutally exclusive or co-occuring with other genes. Signicant differences in survival were found between patients with co-occuring mutations in *RNF43* and *ARID1A* versus patients with mutations in neither (*p* = 0.0454). Significant differnces were also found in patients with co-ocurring mutations in *CDKN2A* and *KRAS* (*p* = 0.0108). Next, no significant differences in survival were found between patients with *KRAS* or *ERBBT* exclusive mutations. Finally, no significant differences in survival were found in patients with co-occuring mutations in *ARID1A* and *KMT2D*, *ARID1A* and *ERBB2*, or *ARID1A* and *PIK3CA* versus wild type.

### 3.10. Comparison of Pancreatobiliary Ampullary Carcinoma vs. Pancreatic Adenocarcinoma

Due to the mophological genomic similarities between pancreatobiliary ampullary carcinoma and pancreatic adenocarcinoma, we investigated these two groups for genomic differences. A summary of the analysis is shown in Table 5.

## 4. Discussion

This study used the AACR Project GENIE database to identify and compare the landscape of somatic mutations in ampullary cancer. Data analysis revealed distinct mutation patterns among differing patient groups.

In agreement with existing literature of national and global incidence patterns, the largest racial cohort was White (*n* = 313) [7,17,18]. Comparative analysis of mutational differences between Whites and Non-Whites revealed differences. The *SLIT2* mutation was exclusively found in the White cohort (*n* = 4). The *PMS1*, *CTNNA1*, *ARHGEF12, SMARCD1*, and *MDC1* mutations were all unique to the Non-White cohort (*n* = 4, 4, 2, 3, 3). While previous research has established that Non-White groups have an overall lower survival rate independent of stage or subtype [8], potential descriptive genetic differences have not been investigated between these population groups. Notably, *CTNNA1*, *SMARCD1*, and *MDC1* mutations occurring in the Non-White cohort are susceptible to epigenetic mutations in prostate, myeloid, and other malignancies [19,20,21,22]. This suggests that inherited susceptivity and socioeconomic factors may intersect in meaningful ways and warrant further exploration in public health [23,24,25,26]. In agreement with the existing literature, this cohort consisted of a majority of males (*n* = 253) and a smaller proportion of females (*n* = 210) [10,17,18]. Comparative analysis between sexes revealed that the mutations in *FANCA* (*n* = 9), *SF3B1* (*n* = 10), and *ATRX* (*n* = 10) were at a higher proportion in females, and the mutations, and *PREX2* (*n* = 11) and *CTNNB1* (*n* = 30) mutations were at higher proportions in males. Notably, the *RARA* mutation (*n* = 6) was seen exclusively in males. These findings suggest that sex-specific molecular mechanisms may underlie ampullary carcinogenesis, highlighting potential for personalized treatment strategies. Histological subtype analysis also revealed noteworthy findings. While distinctions between the intestinal and pancreatobiliary subtypes are well-established and guide current treatment protocols [2,11,13], the mixed subtype—comprising over 40% of ampullary carcinomas [13]—remains poorly understood. This study found *FAT1, RUNX1*, and *ERG* mutations to be more prevalent in the mixed subtype. Although currently untargetable, *FAT1* mutations have been implicated in resistance to epidermal growth factor inhibitors in squamous cell carcinomas [27]. But, when treated with CAMK2 inhibitors concurrently, resistance to treatment decreased [27]. These parallels, though indirect, raise important questions about treatment resistance mechanisms in mixed ampullary cancers and support the need for subtype-specific therapeutic development.

*ATM*, *TP53*, *ARID1A*, and *CDKN2A* are all tumor suppressor genes that are involved in the DNA damage response (DDR) pathway [28,29] and all showed significant frequency in this study’s cohort. Mutations in DDR genes impair DNA-damage sensing and repair, disrupt cell-cycle check points, and reduce apoptosis [30]. Given radiation therapy’s (RT) mechanism of action is to induce DNA damage to cells [31], cancers with these mutations have an increased sensitivity to radiation therapy [32]. Guideline support for radiation therapy in ampullary carcinoma is limited; prospective trials are needed to define indications and timing.

Given recent advances in radiation therapy and the high rate of DDR gene mutations in ampullary cancer, more research is needed to develop personalized RT protocols based on tumor genomics.

Beyond DDR-related genes, co-occurrence was observed in the *ARID1A* mutation with *KMT2D*, *ERBB2*, and *PIK3CA*. It was also observed in the *CDKN2A* with the *SMAD4* and *KRAS* mutations. Thirdly, it was also found in the *TP53* mutation with *CTNNB1*. Lastly, *KRAS* co-occurred with *APC.*

The co-occurrence of these mutations is concurrent with the literature regarding genetic mutations of gastrointestinal carcinomas [33,34]. This broad spectrum of co-occurrences demonstrates the vast heterogeneity that falls within ampullary carcinoma, which has been well documented [33,35].

*ARID1A* and its link to the *KMT2D* mutation are significant because of their implications for therapy targets [36,37]. *ARID1A* is a gene that is involved in chromatin remodeling [34], and *KMT2D* is involved in histone methylation [32]. Their combined mutation suggests epigenetic instability and may confer vulnerability to synthetic-lethality-based therapies like EZH2 inhibitors, PARP inhibitors, and ATR inhibitors [36,38,39]. While the clinical relevance of other co-occurring mutations remains unclear, these findings open avenues development of other targeted therapeutic strategies.

The only mutually exclusive mutations found were *KRAS* and *ERBB2*. This is consistent with one previous study describing the genetic landscape of ampullary cancer in Chinese patients [40]. These findings suggest that either gene may independently drive tumorigenesis and that their co-occurrence could be unfavorable for tumor survival.

This study has several limitations. First, this repository does not include treatment information, limiting the ability to analyze the relationship between treatment responses and mutational status and histologic subtype. Additionally, the absence of therapy data in the database prevents the evaluation of treatment-induced mutational alterations that could complicate comparisons between primary and metastatic tumors. Secondly, the AACR Project GENIE database does not include transcriptomic data. This is important in ampullary carcinoma, where genes—especially those involved in epigenetic regulation—can be overexpressed even without mutations [20,21,22,23]. Without transcriptomic data, it is not possible to correlate mutational status with downstream pathway activity or gene expression levels. Third, the ability to determine significant driver mutations driving pathogenesis from passenger alterations acquired over time was limited by the study design, which did not include a series of samples collected over time with matched analysis. Fourth, though epigenetics was discussed, DNA methylation could not be specifically examined, as this could aid in understanding epigenetic changes. Fifth, in order to include a larger cohort, a majority of analyses were performed aggregating all subtypes (intestinal, pancreatobiliary, mixed, or unknown). Further analysis on mutations can be performed on each subgroup based on sex, race, and age. Sixth, it is acknowledged that there is a confounding effect of having related samples within the cohort (i.e., having many samples from the same patient), though effects are deemed minimal. Seventh, while Kaplan–Meier survival analyses are feasible, the absence of detailed treatment and staging information constrains the interpretation of mutation–survival associations across clinically relevant subgroups. Eighth, as AACR Project GENIE brings data from multiple centers together using different platforms, there may be variabilities in genomic sequencing and estimated rates of mutations. Ninth, because the racial groups other than “White” were so small, margining of all other groups into a “Non-White” group was necessary to provide more statistical power. This grouping limits the ability of the analysis to draw conclusions of the “Non-White” group and radically simplifies the complexity of the cancer. Tenth, because the patient’s age at time of sequencing was used as a proxy for age at diagnosis, potential bias was introduced into the survival analysis: there could be months to years between diagnosis and sequencing. Using age at sequencing as time zero introduces left-truncation and immortal-time bias that underestimate survival, making comparisons between groups less certain. Eleventh, the targeted nature of gene panels can introduce bias, as genes not included on a given panel are reported as wild type despite not being assayed. This limitation is minimized in our study, as analyses focused on the 15 most frequently mutated genes, which were included in nearly all panels across the cohort. Furthermore, mutation frequencies were calculated using the total number of patients as the denominator rather than the number of samples tested per gene, thereby avoiding overestimation for genes assayed in only a limited subset of samples. Lastly, there is a limitation regarding genetic variability and protein expression, as this was not available on the used database. Even with these limitations, the present study still holds steadfast in identifying important genomic profiling in ampullary cancer, highlighting the importance of genetic testing, demographic stratification, mutation profiling, highlighting the uniqueness of the mutational landscape of the mixed histological subtype, and the potential for refinement in radiation therapy and more individualized treatments.

## 5. Conclusions

This study offered a descriptive analysis of somatic mutation frequencies in ampullary carcinoma using data from the AACR Project GENIE database. It identified commonly mutated genes such as *TP53*, *KRAS*, and genes involved in DNA damage response pathways (*ATM*, *TP53*, *ARID1A*, and *CDKN2A*) and reported their distribution across demographic subgroups. In addition, this study noted patterns of co-occurring mutations that may warrant further investigation, including alterations in genes involved in epigenetic regulation. While exploratory in nature, these findings contribute to the growing understanding of the genomic landscape of ampullary carcinoma and may help inform hypotheses for future functional, translational, and clinical research.

## Figures and Tables

**Table 1:** Patient demographics of ampullary carcinoma cohort.

Demographics	Category	*n* (%)
Sex	Male	260 (53.4)
	Unknown	10 (2.1)
	Female	217 (44.6)
Age category at which sequencing was reported	Adult	500 (100)
	Unknown	0 (0)
	Pediatric	0 (0)
Ethnicity	Non-Hispanic	320 (65.7)
	Unknown/Not Collected	106 (21.8)
	Hispanic	61 (12.5)
Race	Asian	40 (8.2)
	White	319 (65.5)
	Black	32 (6.6)
	Other	61 (12.5)
	Unknown	27 (5.5)
Sample Type	Primary	303 (60.6)
	Metastasis	147 (29.1)
	Unspecified/NA	50 (10.0)

**Table 2:** Most Frequent Alterations in Ampullary Cancer Cohort.

Gene	Total Mutations (%)	Samples with ≥1 Mutation (%)
*TP53*	266 (53.2)	247 (49.4)
*KRAS*	233 (46.6)	228 (45.6)
*SMAD4*	83 (16.6)	78 (15.6)
*APC*	108 (21.6)	83 (16.6)
*PIK3CA*	52 (10.4)	50 (10.0)
*ARID1A*	60 (12.0)	45 (9.0)
*CDKN2A*	46 (9.2)	44 (8.8)
*KMT2D*	56 (11.2)	40 (8.0)
*CTNNB1*	43 (8.6)	42 (8.4)
*ERBB2*	44 (8.8)	34 (6.8)
*ATM*	36 (7.2)	34 (6.8)
*RNF43*	34 (6.8)	29 (5.8)
*ERBB3*	33 (6.6)	27 (5.4)

**Table 3:** Differences in Mutations by Sex and Race. Denominator is profiled samples.

Gene	Male, *n* (%)	Female, *n* (%)	*p* Value	*q* Value
*FANCA*	1 (0.43)	10 (5.35)	3.86 × 10^−4^	0.116
*SF3B1*	3 (1.25)	10 (5.13)	2.29 × 10^−2^	0.444
*ATRX*	3 (1.29)	10 (5.35)	2.21 × 10^−2^	0.440
*PREX2*	11 (7.69)	2 (1.82)	4.41 × 10^−2^	0.748
*CTNNB1*	31 (11.61)	12 (5.41)	1.64 × 10^−2^	0.440
*RARA*	5 (2.18)	0 (0.0)	6.92 × 10^−2^	1.00
	**White, *n* (%)**	**Non-White, *n* (%)**	***p* Value**	***q* Value**
*TP53*	183 (55.5)	50 (37.0)	3.43 × 10^−4^	0.180
*KRAS*	170 (51.5)	45 (33.3)	4.71 × 10^−4^	0.180
*LRP1B*	10 (22.7)	1 (1.82)	2.09 × 10^−3^	0.216
*SOX9*	22 (8.18)	2 (1.60)	1.13 × 10^−2^	0.319
*IRS2*	15 (6.61)	1 (0.86)	1.47 × 10^−2^	0.389
*SMAD4*	67 (20.36)	15 (11.11)	2.22 × 10^−2^	0.471

**Table 4:** Mutations Correlated with Reduced Survival.

Mutation	Differential Survival	Number of Patients with Wild Type	Number of Patients with Mutation	*p* Value	Gene Function (GeneCards)
*TP53*	Reduces Survival	209	208	0.071	Tumor suppressor
*KRAS*	Reduces Survival	231	186	0.019	Ras family oncogene

**Table 5:** Differences in mutations in Pancreatobiliary Ampullary Carcinoma vs. Pancreatic Adenocarcinoma.

Gene (Chi-Squared)	Pancreatobiliary Ampullary Carcinoma	Pancreatic Adenocarcinoma	*p* Value	*q* Value	Enriched In
*KRAS*	80 (50.63%)	7215 (77.81%)	<10 × 10^−10^	<10 × 10^−10^	Pancreatic Adenocarcinoma
*PIK3CA*	14 (8.81%)	263 (2.84%)	2.370 × 10^−4^	4.577 × 10^−3^	Pancreatobiliary Ampullary Carcinoma
*ERBB2*	14 (8.86%)	221 (2.38%)	3.874 × 10^−5^	8.290 × 10^−4^	Pancreatobiliary Ampullary Carcinoma
*BAP1*	7 (4.86%)	82 (0.97%)	7.086 × 10^−5^	1.28 × 10^−2^	Pancreatobiliary Ampullary Carcinoma
*ATM*	16 (10.46%)	392 (4.34%)	1.239 × 10^−3^	0.0212	Pancreatobiliary Ampullary Carcinoma

## Data Availability

The data presented in this study are openly available in cBioPortal for GENIE at Available online: https://genie.cbioportal.org (accessed on 17 October 2025), and cBioPortal for GENIE at Available online: https://genie.cbioportal.org/?continue (accessed on 17 October 2025).

## Supplementary Materials

The following supporting information can be downloaded at: https://www.mdpi.com/article/10.3390/cimb47110932/s1.
