# Supplementary material for: Descriptive Genomic Analysis of Ampullary Carcinoma Utilizing the AACR Project GENIE Dataset
Source: Curr Issues Mol Biol. 2025 Nov 9;47(11):932. doi: 10.3390/cimb47110932 (PMC12651240; doi:10.3390/cimb47110932)
Supplement: Supplementary file 1 [file cimb-47-00932-s001.zip › cimb-3899915-supplementary.pdf]

Table S1. Mutations by Histological Subcategory.

Gene (Chi-Squared)	Not subcategorized, n (%)	Intestinal, n (%)	Pancreatobiliary, n (%)	Mixed, n (%)	*p* Value	*q* Value	*Enriched In*
APC	52 (18.77)	20 (55.56)	9 (5.84)	2 (16.67)	$< 10\ e^{-10}$	$7.84e^{-10}$	*Intestinal*
CTNNB1	27 (9.38)	9 (23.68)	7 (4.43)	1 (6.67)	$2.379e^{-3}$	*0.0186*	*Intestinal*
SOX9	15 (6.41)	10 (33.33)	2 (1.40)	2 (18.18)	$2.329e^{-9}$	$8.16e^{-8}$	*Intestinal*
FAT1	12 (5.83)	3 (10.34)	4 (4.21)	4 (36.36)	$6.470\ e^{-4}$	$5.823\ e^{-3}$	*Mixed*
RUNX1	3 (1.28)	0 (0.00)	0 (0.00)	2 (16.67)	$3.208e^{-5}$	$3.441e^{-4}$	*Mixed*
ERG	4 (1.78)	1 (3.33)	0 (0.00)	2 (18.18)	$1.213e^{-4}$	$1.266e^{-3}$	*Mixed*
BAP1	2 (0.83)	1 (3.13)	7 (4.86)	0 (0.00)	*0.0479*	$7.57e^{-2}$	*Pancreatobiliary*
